# Perioperative Management of a Patient With Very Long Chain Acyl-CoA Dehydrogenase Deficiency Undergoing Laparoscopic Sleeve Gastrectomy: First Report of Bariatric Surgery in VLCADD

**DOI:** 10.1007/s11695-025-08347-w

**Published:** 2025-12-05

**Authors:** Mirza Anwar Baig, Bennedict Williams

**Affiliations:** University Hospitals of North Midlands NHS Trust, Stoke-on-Trent, United Kingdom

**Keywords:** VLCADD, Bariatric surgery, Rhabdomyolisis, Hypoglycemia, Nutritional management

## Abstract

Very Long Chain Acyl-CoA Dehydrogenase Deficiency (VLCADD) is a rare inherited disorder of mitochondrial fatty acid β-oxidation that predisposes patients to metabolic crises, rhabdomyolysis, and cardiomyopathy. Surgical stress, fasting, and anaesthesia may precipitate metabolic decompensation. We present the case of a 65-year-old female with late-onset VLCADD, multiple comorbidities, and chronic kidney disease (CKD) stage 3 who underwent laparoscopic sleeve gastrectomy. Despite perioperative glucose infusion, normothermia, and careful anaesthetic planning, she developed postoperative rhabdomyolysis. This case highlights perioperative challenges in VLCADD, provides practical strategies for anaesthetic and nutritional management, and, to our knowledge, represents the first report of bariatric surgery in a patient with VLCADD. Long-term follow-up demonstrated sustained weight loss, improved cardiometabolic profile, and stable muscle and renal function.

## Introduction

Very Long Chain Acyl-CoA Dehydrogenase Deficiency (VLCADD) is an autosomal recessive disorder results from pathogenic variants in ACADVL, leading to impaired mitochondrial oxidation of long-chain fatty acids (C14–C20). Three phenotypes are recognised: (i) a severe infantile form with cardiomyopathy andhigh mortality, (ii) a childhood hypoketotic-hypoglycaemic form, and (iii) an adult myopathic form characterised by exertional myalgia and rhabdomyolysis [1]. In a patient with VLCAD deficiency, as β-oxidation is impaired, ATP is not normally produced from fatty acids in the liver and muscles. Significant ATP deficiency in muscles results in rhabdomyolysis [2]. Patients are vulnerable to metabolic decompensation during catabolic stress, including fasting, infection, or surgery. Anaesthetic management is particularly challenging due to risks of rhabdomyolysis, metabolic acidosis, and renal injury. Bariatric surgery is rarely described in this population. To our knowledge, this is the first published case of bariatric/metabolic surgery in VLCADD.

## Case Presentation

A 65-year-old female with a BMI of 46.1 kg/m² was scheduled for laparoscopic sleeve gastrectomy. Her past history included late-onset VLCADD, hypertension, type 2 diabetes mellitus, gastroesophageal reflux disease, osteoarthritis, a non-specific cardiac condition, and CKD stage 3 (baseline eGFR 45 mL/min/1.73 m²). She reported previous perioperative exacerbations of VLCADD with myalgia and elevated creatine kinase (CK). Preoperative CK was 280 U/L (normal < 170).

Given her comorbidities and metabolic disorder, a multidisciplinary team (MDT) involving bariatric surgery, anaesthesia, dietetics, internal medicine, and cardiology reviewed her. Bariatric surgery was deemed appropriate because lifestyle interventions were limited by exercise intolerance, myalgia and comorbidities. Preoperative planning emphasised avoidance of fasting, perioperative glucose infusion, and careful anaesthetic selection. She was started on liver shrinkage 2 weeks prior to the surgery and to be followed for 6 weeks post surgery. She was followed by a a specialised center for Inherited Metabolic disorders for her diet and nutritional requirements.

## Preoperative Evaluation and Planning

Preoperative evaluation revealed CKD stage 3 (eGFR 45 mL/min/1.73 m²) and ECG with evidence of an old inferior infarct; echocardiogram was normal. Nutritional strategy included minimising fasting, carbohydrate drinks up to 2 h before surgery, and initiation of IV 10% dextrose with variable rate insulin infusion (VRII). Active warming was planned. Anaesthetic plan avoided propofol, favouring remifentanil and volatile agents. The patient sustained a minor fall pre-operatively but CT brain was normal. She was counselled and consented for general anaesthesia with a plan to observe her post operatively in Surgical High dependency unit.

## Intraoperative Management

Standard AAGBI monitoring with invasive arterial pressure and BIS was applied. Induction was achieved with remifentanil infusion, midazolam 2 mg, and sevoflurane inhalation. Propofol was avoided. Rocuronium was used for intubation, reversed with sugammadex. Maintenance included remifentanil target-controlled infusion and sevoflurane to MAC of 1. Temperature was maintained 36.5–37 °C with forced air warming and warmed IV fluids. Intraoperative blood glucose remained 5–9 mmol/L. ABG was unremarkable.

## Postoperative Course and Complications

In PACU she was haemodynamically stable but CK rose to 7300 U/L, consistent with rhabdomyolysis. She was actively warmed to prevent shivering. She was transferred to surgical HDU. Management included aggressive IV fluids to prevent pigment nephropathy, VRII with glucose, electrolyte and renal monitoring, and multimodal analgesia. CK gradually declined to minimum of 2045 on discharge at 5th post op day. Creatinine remained stable (baseline 110 µmol/L → peak 120 µmol/L; eGFR 45 → 42). Potassium and phosphate were within normal limits. No myoglobinuria was noted.

## Follow-up

She was readmitted 2 weeks later with poor oral intake and myalgia. She was not happy with the post op diet and the recovery overall. She was febrile with a max temperature reading of 38.9 °C. Creatinine kinase at the time of readmission was 13,402 U/L. A diagnosis of UTI was made. She remained in the hospital for another 1 week and was treated with antibiotics, her blood sugars managed with VRII and received appropriate supportive therapy. She was discharged 1 week later after her symptoms were effectively managed, creatinine kinase came down to 3540 U/L and her blood sugars were within normal range.

At 3 and 8months follow-up, CK remained < 300 U/L, creatinine 95–100 µmol/L (eGFR ~ 50 mL/min/1.73 m²), and no rhabdomyolysis episodes occurred. Weight reduction achieved was 25.4% of the pre op. Diabetes control improved (HbA1c 8.5% → 6.9%), she is off oral hypoglycemics, hypertension medications were reduced, and OSA symptoms improved. She was able to mobilise in the house with support and mood appeared better with better tolerance to post surgery dietary plans. No cardiovascular or metabolic crises were reported.

## Discussion

VLCADD poses unique perioperative risks due to impaired long-chain fatty acid oxidation. Catabolic stress, fasting, and hypothermia can precipitate rhabdomyolysis and metabolic crises in these patients. Key management principles include minimising fasting, providing continuous glucose, maintaining normothermia, and careful anaesthetic drug selection [3]. Volatile anaesthetics (sevoflurane, desflurane) are generally safe and widely reported in FAOD cases. Propofol is avoided in prolonged infusions (risk due to lipid carrier and mitochondrial inhibition). Small boluses for induction may be tolerated, but many centres prefer alternative induction agents. Remifentanil is preferred (short acting, rapid clearance, minimal metabolic load) opioid. Use non-depolarising agents (e.g., rocuronium, atracurium) for muscle relaxations. We have to avoid succinylcholine as there is a risk of hyperkalaemia and rhabdomyolysis [4]. Bariatric surgery is not a contraindication in VLCADD. Bariatric procedures (especially Roux-en-Y gastric bypass and sleeve gastrectomy) induce rapid weight loss and caloric restriction, which can trigger catabolism. In fatty oxidation disorders, this may precipitate hypoglycemia, rhabdomyolysis, and metabolic crises [5]. These patients requires tailored nutritional management and careful monitoring of glucose, creatinine kinase and liver enzymes [6]. Patients for bariatric surgery require to be on high protein and low calorie diet [7]. But our patient was put on a diet in consultation with a centre specialising in Inherited Metabolic Disorders. There was plan for moderate calorie restriction (not below 1200 kcal/day). She was prescribed High complex carbohydrate intake to maintain glycogen stores. She was advised low long-chain fat intake, but include essential fatty acids and Medium-chain triglycerides (MCTs) were prescribed if tolerated. Adequate protein intake (1.0–1.2 g/kg/day) to support lean mass. Serial measurements of blood glucose, creatinine kinase, liver enzymes is required to detect any metabolic crisis and manage them appropriately [8].

Our case highlights the importance of early MDT involvement, dietetic input, and structured perioperative pathways. To our knowledge, this is the first report of bariatric surgery in VLCADD. Despite early postoperative rhabdomyolysis, aggressive hydration and metabolic support prevented acute kidney injury, and long-term outcomes were favourable (Table 1).

**Table 1:**
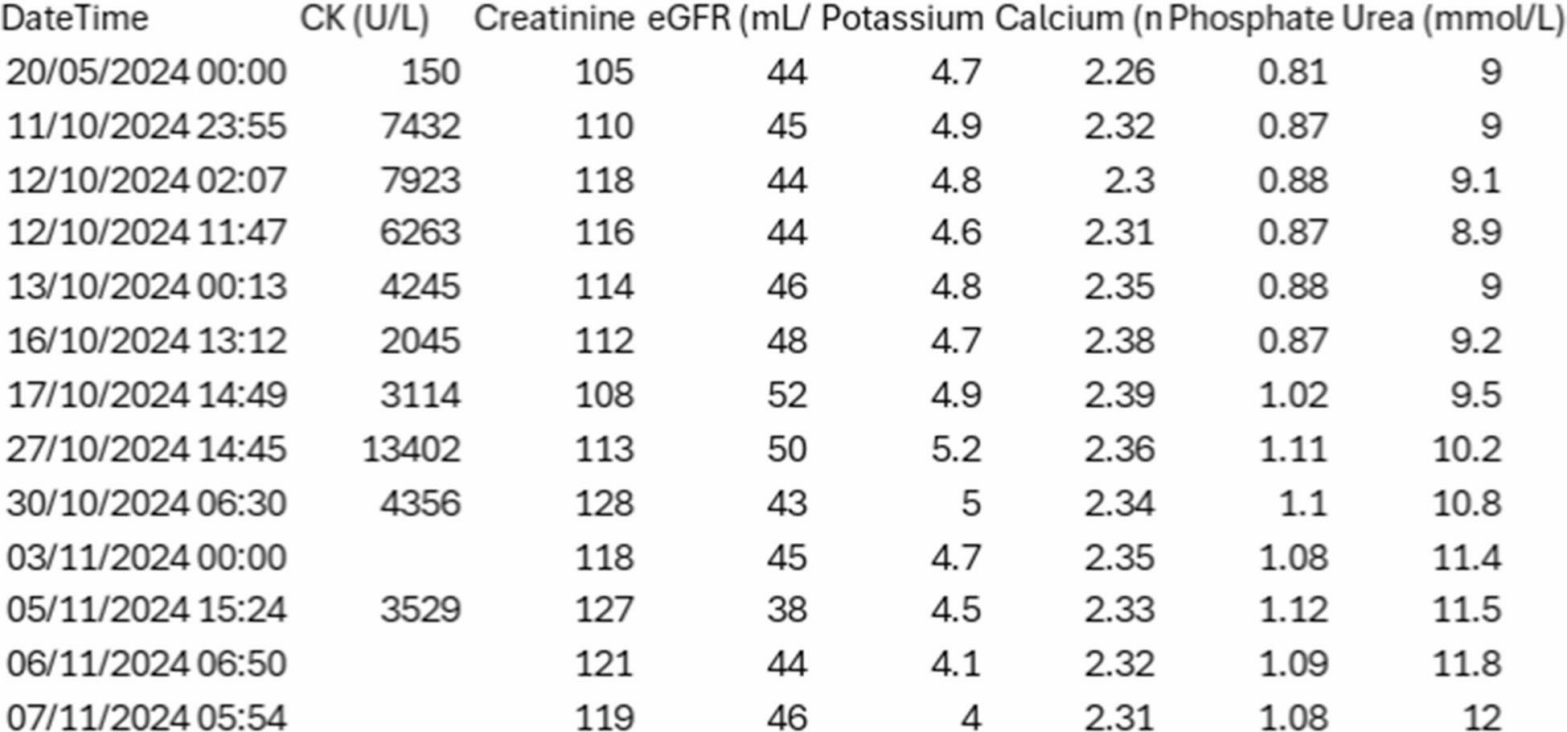
Perioperative laboratory results as derived from the internal reporting system

*CK* creatinine, *eGFR* urea, *K⁺, Ca²⁺* phosphate, *AST/ALT* lactate at baseline, 24 h, 48 h, discharge, and follow-up.

## Conclusion

This case demonstrates that bariatric surgery can be performed safely in patients with VLCADD when guided by a structured, multidisciplinary, and disease-specific perioperative plan. Key measures include avoidance of fasting, perioperative glucose infusion, maintenance of normothermia, and close biochemical monitoring. Our report expands the literature by documenting the first bariatric case in VLCADD and provides practical lessons for anaesthetic and perioperative teams.

## Data Availability

No datasets were generated or analysed during the current study.
